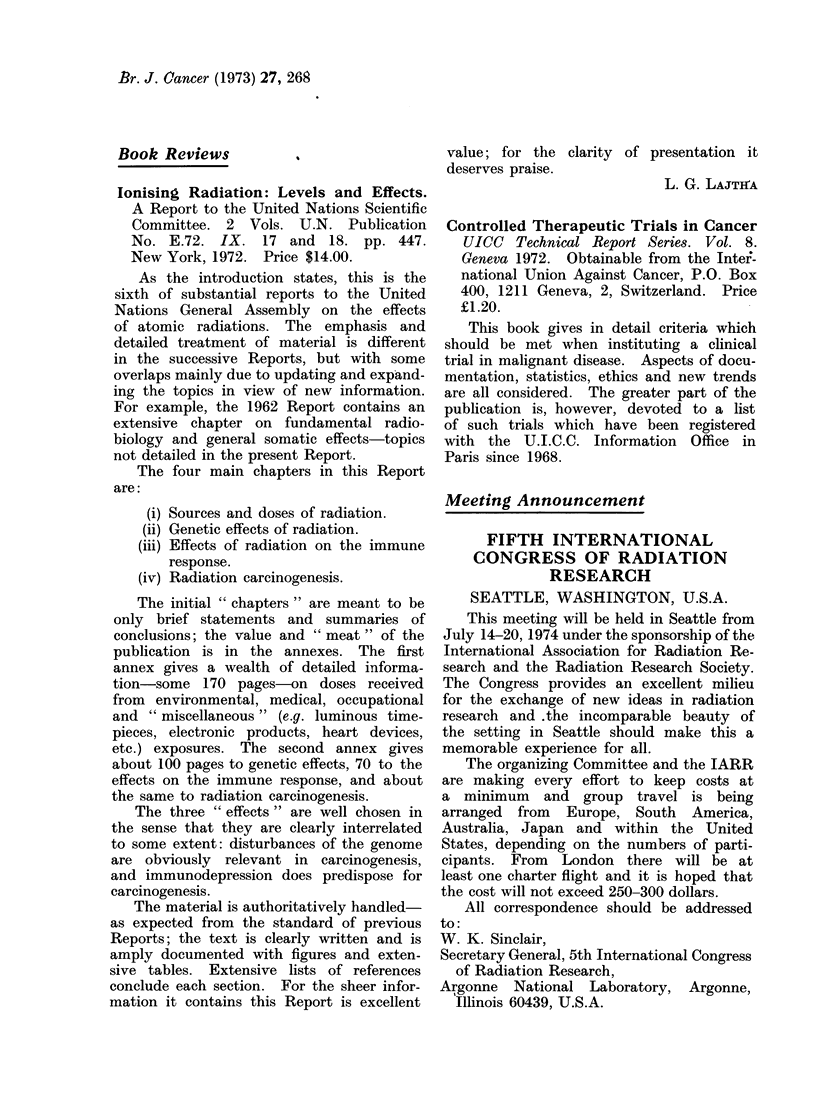# Controlled Therapeutic Trials in Cancer

**Published:** 1973-03

**Authors:** 


**Controlled Therapeutic Trials in Cancer** *UICC Technical Report Series. Vol.* 8. *Geneva* 1972. Obtainable from the International Union Against Cancer, P.O. Box 400, 1211 Geneva, 2, Switzerland. Price £1.20.

This book gives in detail criteria which should be met when instituting a clinical trial in malignant disease. Aspects of documentation, statistics, ethics and new trends are all considered. The greater part of the publication is, however, devoted to a list of such trials which have been registered with the U.I.C.C. Information Office in Paris since 1968.